# Relationship between religiosity and HPV vaccine initiation and intention in urban black and hispanic parents

**DOI:** 10.1186/s12889-024-17653-4

**Published:** 2024-01-23

**Authors:** Deidra Carroll Coleman, Christine Markham, Vincent Guilamo-Ramos, Diane Santa Maria

**Affiliations:** 1 Department of Health Disparities Research, The University of Texas MD Anderson Cancer Center, 1400 Pressler St., Unit. 1440, 77030 Houston, TX USA; 2 Department of Health Promotion and Behavioral Sciences, The University of Texas Health Science Center at Houston, School of Public Health, 77030 Houston, TX USA; 3 Center for Latino Adolescent and Family Health, Duke University School of Nursing, 27710 Durham, MC USA; 4 Department of Research, The University of Texas Health Science Center at Houston, Cizik School of Nursing, 77030 Houston, TX USA

**Keywords:** African American, Healthcare disparities, Hispanic, Human papillomavirus, HPV vaccine, Religion

## Abstract

**Objective:**

Religion is believed to be an important sociocultural influence in the U.S., but little is known about how religiosity shapes the human papillomavirus (HPV) vaccine decision in racial/ethnic minorities. The purpose of this study was to examine the relationship between religiosity and HPV vaccine initiation and intention among urban, racial/ethnic minority parents of adolescents 11–14 years old.

**Design:**

This study employed a descriptive, cross-sectional design using baseline data from Black and Hispanic parents (*N* = 175 and 285, respectively) recruited from medically underserved communities. Chi-square tests for independence and independent-samples t-tests were run to assess sociodemographic differences in vaccine initiation and vaccine intention. Binary logistic regression analyses were conducted to determine whether religious attendance and religious salience were associated with parents’ HPV vaccine decisions for their children.

**Results:**

Approximately 47% of Black parents had vaccinated their youth against HPV. Of those who had not initiated the vaccine for their child, 54% did not intend to do so. 54% of Hispanic parents had initiated the HPV vaccine for their youth. Of those who had not initiated the vaccine for their child, 51% did not intend to do so. Frequency of attendance at religious services and the importance of religion in one’s life was not significantly correlated with HPV vaccine decision-making for Black nor Hispanic parents.

**Conclusion:**

This study suggests that religiosity does not influence the HPV vaccine decision for urban, Black and Hispanic parents. Future studies using measures that capture the complexity of religion as a social construct are needed to confirm the findings. In addition, studies with representative sampling will enable us to make generalizations about the influence of religion on HPV vaccine decision-making for urban, racial/ethnic minority parents.

Human papillomavirus (HPV), the most common sexually transmitted infection in the United States (U.S.), causes nearly all cervical cancer and many cancers of the vagina, vulva, penis, anus, and throat [1]. More than 47,000 HPV-associated cancers are diagnosed in the U.S. annually, and racial/ethnic minorities bear a disproportionate burden of these diagnoses [2]. Additionally, Black and Hispanic women have the highest cervical cancer mortality rate of all racial/ethnic groups [3]. These differences exist for several reasons, including low access to preventive services, late-stage diagnoses, and decreased access to follow-up treatment [4–6]. However, increasing HPV vaccine coverage rates for Black and Hispanics could reduce disparities in HPV-associated morbidity and mortality.

The CDC estimates that, in the state of Texas, only 38% of Black youth and 45% of Hispanic youth are up-to-date on the HPV vaccine [7]. Black and Hispanic parents report several barriers to vaccinating youth against HPV, including a lack of recommendation from healthcare providers, concerns about vaccine safety and efficacy, and low perceived risk of children contracting HPV [8–10]. In an integrative review to determine factors associated with vaccine acceptance and initiation among racial/ethnic minority parents, researchers found that religiosity was also a barrier to HPV vaccine initiation [11]. However, at the time of the review, only three studies examining the relationship between religiosity and the HPV vaccine decision among Black and Hispanic parents had been conducted. Two of these studies focused on parents living in rural areas [12–13] and the third study excluded parents of sons [14].

The extent to which religiosity is related to the HPV vaccine decision for Black and Hispanic parents deserves further examination. Black and Hispanic people are the most religious racial/ethnic groups in the U.S. For example, 47% of Black adults and 39% Hispanic adults report attending religious services at least once a week, compared to 35% of non-Hispanic white adults [15]. Shelton et al. (2013) found that parents who frequently attended religious services were more likely to have decided against the HPV vaccination for their 9–13 year old children and to believe that no children should receive the HPV vaccine [14]. Similarly, Barnack et al. (2010) and Constantine & Jerman (2007) reported a negative relationship between religious attendance and parents’ intention to vaccinate their daughters against HPV [16–17]. In contrast, Litton et al. (2012) found that religious attendance did not impact parents’ intent to vaccinate their children against HPV [18]. These mixed findings obscure our understanding of the relation between religious attendance and the HPV vaccine decision.

In addition to attending religious services more frequently than other racial/ethnic groups, Black and Hispanic adults report greater religious salience (i.e., the degree to which religion is important in life). More specifically, 75% of Black adults and 59% of Hispanic adults report that religion is ‘very important’ in their daily life, compared to 49% non-Hispanic white adults [18]. Sadigh et al. (2012) found that mothers who reported that religion was highly important in their lives had lower odds of vaccinating their 9–18 year old daughters [19]. This finding is consistent with the findings of Reiter et al. (2010) which demonstrated that mothers who reported high levels of religious importance were less willing to vaccinate their sons [20]. These studies suggest that religious salience may be a barrier to HPV vaccine initiation.

As noted above, few studies have focused on the relationship between religion and the HPV vaccine decision among racial/ethnic minority populations. Lahijani et al. (2021) conducted focus groups with church leaders, parents of 9–17 year olds, and young adults ages 18 to 26 years who were part of a large African Methodist Episcopal church [21]. Participants reported a wide range of perceptions regarding the vaccine (i.e., unnecessary, “sex-permitting,” unsure, “better safe than sorry,” and necessary). Additionally, Galbraith-Gyan et al. (2018) and Thompson et al. (2013), in their interviews with Black parents, found that among parents who had and had not initiated the HPV vaccine for their daughters, religion was not a factor [22–23]. These studies suggest that for at least some Black parents, religion is not a barrier to vaccinating their youth against HPV; however, qualitative studies cannot help us understand trends in HPV vaccine initiation for the target population. Furthermore, no studies have examined the extent to which religiosity is related to the HPV vaccine decision of Hispanic parents.

To determine the relationship between religiosity, HPV vaccine initiation, and HPV vaccine intention among racial/ethnic minorities, we conducted a cross-sectional study with a community-based sample of Black and Hispanic parents of 11 to 14 year old girls and boys. Here we operationalized religiosity as religious attendance and religious salience since these definitions have been used in previous research and will help us situate study findings in the broader literature. This study extends that literature by focusing on the vaccine behavior and intentions of urban, Black and Hispanic parents in particular.

## Methods

The current study is a secondary analysis of baseline data collected for a randomized controlled trial (RCT) designed to test the effects of a parent-based, adolescent sexual health intervention [24]. In the broader study, a convenience sampling strategy was used to recruit 11–14 year old youth and their caregivers from 21 after-school programs (e.g., Boys and Girls Club) and 19 charter schools in medically underserved communities. Study flyers were posted at each recruitment site and representatives from after-school programs and charter schools disseminated recruitment information to eligible families either in-person, by email, or using robo-calls. Additionally, research personnel hosted recruitment events at study sites and recruited participants at community-based events (e.g., Parent-Teacher Organization meetings, school yard sales, health fairs). For inclusion in the study, caregivers (herein referred to as “parents”) had to be living with a 11–14 year old youth and self-identify as either Hispanic (any race) or non-Hispanic Black. Parents were included in the sample regardless of their group assignment in the broader study, since baseline assessments were completed prior to intervention delivery. The response rate at baseline was 93%. All study procedures were approved by the ethics Committee for the Protection of Human Subjects (HSC-SN-15-0091) at a public academic health science center in the southern U.S. Written informed consent was obtained from all participants.

### Data collection

Data collection took place between fall 2015 and spring 2018 through an online self-administered survey. Baseline surveys captured sociodemographic data for parents, including sex, race/ethnicity, education level, annual household income, insurance status, and religiosity.

**Religious Factors**– To assess frequency of attendance at religious services (*religious attendance*), which provides meaning about religious involvement, parents were asked, ‘How often have you gone to religious services in the past 12 months?’ (never; once or twice a year; every month or so; once or twice a month; once a week; more than once a week). The second question assessed the relative importance of religion in one’s personal life (*religious salience*), and is a proxy for the extent to which religion shapes one’s behavior. Parents were asked, ‘How important is religion in your life?’ (not at all important; somewhat important; important; very important). Single-item measures of religious attendance and religious salience have been used to assess religiosity in previous research on the HPV vaccine decision [14, 16, 17].

**HPV Vaccine Decision**– To assess whether parents had started the vaccine series for their 11–14 year old youth (*vaccine initiation*), parents were asked, ‘Did your child receive dose 1 of the 3-dose HPV vaccine series?’ (yes or no). To assess whether parents who had not yet initiated the vaccine series for their youth intended to do so in the future (*vaccine intention*), parents were asked ‘Are you planning to give your child all 3 doses of the HPV vaccine series?’ (yes or no).

### Data analysis

Descriptive statistics were used to summarize the sociodemographic characteristics of the sample. Chi-square tests for independence and independent-samples t-tests were run to assess sociodemographic differences in vaccine initiation and vaccine intention. Binary logistic regression analyses were performed to determine whether various levels of religious attendance and religious salience were associated with vaccine initiation and vaccine intention. The outcome variable, vaccine initiation, was coded 0 = has not received any doses of the HPV vaccine series, and 1 = has received dose 1 of the 2 or 3 dose HPV vaccine series. Similarly, vaccine intention was coded 0 = does not intend to give child all 2 or 3 doses of the HPV vaccine series, and 1 = intends to give child all 2 or 3 doses of the HPV vaccine series. Note that during the study period, the recommendation changed from 3 to 2 vaccines for certain youth in this age range. We modeled outcomes for Black and Hispanic parents separately since little is known about the extent to which religiosity is related to the HPV vaccine decision for these racial/ethnic groups, and given the unique ways culture may shape their vaccine behavior. This decision was made prior to analyses. Statistical analyses were conducted using IBM SPSS, v. 29.0.

## Results

A total of 519 parents were enrolled in the RCT of which 175 were Black and 285 were Hispanic. Parents in this secondary analysis were primarily female, had a total annual household income of $49,999 or less, and had Medicaid or no insurance coverage (Table 1). In addition, most parents identified as Christian (93.2%), attended services once a month or more frequently (67.2%), and reported that religion is very important in their life (54.8%). When compared to Black parents, a greater proportion of Hispanic parents had initiated the HPV vaccine for their youth (47.4% vs. 54.4%).

**Table 1:** Demographic characteristics, HPV vaccine intention and initiation by race/ethnicity

	Total (*n* = 460)		Black (*n* = 175)		Hispanic (*n* = 285)	
	N	%	n	%	n	%
Parent gender						
Male	45	9.8	21	12.0	24	8.4
Female	415	90.2	154	88.0	261	91.6
Education						
HS diploma or less	195	42.4	36	20.6	159	55.8
Vocational/technical/some college	166	36.1	78	44.6	88	30.9
College degree	99	21.5	61	34.8	38	13.3
Income						
Less than $25,000	179	38.9	56	32.0	123	43.2
$25,000 to $49,999	197	42.8	79	45.1	118	41.4
$50,000 or more	83	18.3	40	22.9	43	15.1
Insurance						
None	75	16.3	19	10.9	56	19.6
Medicaid	191	41.5	56	32.0	135	47.4
Private	171	37.2	94	53.7	77	27.0
Other	51	11.0	19	10.9	32	11.2
Religious tradition						
Christian	429	93.3	163	93.1	266	93.3
Other religion	11	2.4	2	1.2	9	3.2
None	20	4.3	10	5.7	10	3.5
Religious attendance						
Never	39	8.5	18	10.3	21	7.4
Once or twice a year	58	12.6	27	15.4	31	10.9
Every month or so	54	11.7	22	12.6	32	11.2
Once or twice a month	87	18.9	35	20.0	52	18.2
Once a week	136	29.6	42	24.0	94	33.0
More than once a week	86	18.7	31	17.7	55	19.3
Religious salience						
Not at all important	13	2.8	5	2.9	8	2.8
Somewhat important	55	12.0	46	9.1	39	13.7
Important	140	30.4	40	22.9	100	35.1
Very important	252	54.8	114	65.1	138	48.4
Child gender						
Boy	216	47.0	79	45.1	137	48.1
Girl	224	48.7	87	49.7	137	48.1
HPV vaccine initiation						
Initiated the vaccine	238	51.7	83	47.4	155	54.4
Has not initiated the vaccine	22	48.3	92	52.6	130	45.6
HPV vaccine intention						
Intends to vaccinate	106	47.7	42	45.7	64	49.2
Does not intend to vaccinate	116	52.3	50	54.3	66	50.8

HPV: human papillomavir

### Black parents

Approximately 47% of Black parents had initiated the HPV vaccine series for their youth. Females were more likely to have initiated the series for their youth than males (χ^2^ = 5.34, *df* = 1, *p* =.02, data not shown), but no other demographic differences were found between parents who had and had not initiated the vaccine series. 46% of the parents who had not initiated the HPV vaccine series at baseline reported intending to do so in the future. There were no demographic differences between parents who did and did not intend to vaccinate their youth against HPV.

Bivariate associations between religiosity, HPV vaccine initiation, and HPV vaccine intention are presented in Table 2. A total of 175 and 92 Black parents were included in the HPV vaccine initiation and HPV vaccine intention models, respectively. Frequency of attendance at religious services was not significantly correlated with the youth receiving dose 1 of the three dose vaccine series or parents’ intention to vaccinate their youth. Similarly, religious salience was not significantly associated with parents initiating the HPV vaccine series for their youth or their intention to vaccinate youth against HPV.

**Table 2:** Binary logistic regression analysis for the association between religious factors and HPV vaccine initiation and intention for Black parents

	Initiated Vaccine Series (*n* = 175) OR (95% CI)	*p*-value	Intends to Vaccinate (*n* = 92) OR (95% CI)	*p*-value
*Religious Attendance*				
Never	(ref)		(ref)	
Once or twice a year	1.18 (0.34, 4.12)	0.80	0.43 (0.09, 2.14)	0.30
Every month or so	3.50 (0.95, 12.97)	0.06	1.40 (0.23, 8.46)	0.71
Once or twice a month	1.18 (0.36, 3.91)	0.78	1.40 (0.34, 5.79)	0.64
Once a week	2.67 (0.84, 8.46)	0.10	1.75 (0.40, 7.66)	0.46
More than once a week	2.13 (0.64 7.13)	0.22	1.60 (0.35, 740)	0.55
*Religious Salience*				
Not at all important	(ref)		(ref)	
Somewhat important	1.50 (0.19, 11.54)	0.70	0.00 (0.00, 0.00)	1.00
Important	1.83 (0.28, 12.19)	0.53	0.00 (0.00, 0.00)	1.00
Very important	1.21 (0.20, 7.55)	0.84	0.00 (0.00, 0.00)	1.00

Note: Models adjusted for parents’ gender, education, income, religious tradition, child’s gender, and child’s age; OR: odds ratio; CI: confidence interval

### Hispanic parents

54% of Hispanic parents had initiated the HPV vaccine series for their youth. Females were more likely to have initiated the HPV vaccine for their youth than males (χ^2^ = 9.12, *df* = 1, *p* =.00, data not shown), and parents were more likely to have initiated the vaccine for their daughters than for their sons (χ^2^ = 5.30, *df* = 1, *p* =.02). We found no significant differences in vaccine initiation by parents’ level of education, income, years living in the U.S., religious tradition, or child’s age. Additionally, 49% of the parents who had not initiated the vaccine series for their child, reported intending to do so in the future. Parents who intended to vaccinate their youth against HPV were more likely to earn $25,000 or less annually (χ^2^ = 13.97, *df* = 2, *p* =.00).

The bivariate relationship between religiosity, HPV vaccine initiation, and HPV vaccine intention are presented in Table 3. A total of 285 and 130 Hispanic parents were included in the HPV vaccine initiation and HPV vaccine intention models, respectively. Frequency of attendance at religious services was not significantly associated with initiating the HPV vaccine series for youth or planning to vaccinate youth against HPV. Furthermore, the importance of religion in parents’ life was not significantly correlated with youth having received the first dose of the HPV vaccine series or parents’ intention to vaccinate youth against HPV.

**Table 3:** Binary logistic regression analysis for the association between religious factors and HPV vaccine initiation and intention for Hispanic parents

	Initiated Vaccine Series (*n* = 285) OR (95% CI)	*p*-value	Intends to Vaccinate (*n* = 130) OR (95% CI)	*p*-value
*Religious Attendance*				
Never	(ref)		(ref)	
Once or twice a year	1.29 (0.41, 4.12)	0.66	1.50 (0.23, 9.80)	0.67
Every month or so	0.62 (0.20,1.89)	0.40	1.00 (0.18, 5.46)	1.00
Once or twice a month	0.84 (0.30, 2.37)	0.74	1.75 (0.34, 8.98)	0.50
Once a week	0.70 (0.27, 1.84)	0.47	1.10 (0.24, 4.94)	0.91
More than once a week	0.51 (0.18, 1.43)	0.20	0.43 (0.09, 2.10)	0.30
*Religious Salience*				
Not at all important	(ref)		(ref)	
Somewhat important	0.75 (0.13, 4.27)	0.75	1.00 (0.05, 19.96)	1.00
Important	0.41 (0.08, 2.12)	0.29	1.50 (0.09, 25.55)	0.80
Very important	0.32 (0.06, 1.61)	0.17	0.73 (0.04, 12.17)	0.83

Note: Models adjusted for parents’ gender, education, income, religious tradition, child’s gender, and child’s age; OR: odds ratio; CI: confidence interval

## Discussion

We found that frequency of attendance at religious services and the importance of religion in one’s life were not associated with HPV vaccine initiation or intention for this community-based sample of Black and Hispanic parents. These findings align with those from qualitative studies suggesting that religiosity does not impact Black parents’ vaccine decisions [21–23] and provide novel data on Hispanic parents’ vaccine behavior. The results of the current study contrast with empirical evidence suggesting that religiosity is a barrier to parents’ HPV vaccine decisions broadly [14, 16–17, 19–20], and for racial/ethnic minority parents in particular [11]. This divergence may point to racial/ethnic differences in the former case and geographical differences in the latter, since previous studies focused on rural parents.

Our findings have important implications for how healthcare and public health practitioners approach HPV vaccine administration and education. For example, if religiosity is not a barrier to HPV vaccine uptake, partnering with religious leaders on HPV vaccine programs and events may be strategic. The Black church has historically offered programming and services that focus on health issues central to the Black community [25], and for Hispanic families, the church is a preferred site for receiving health information [26]. In addition, informing healthcare providers that religious Black and Hispanic parents are no less likely than the non-religious to vaccinate their children against HPV may be important. Cataldi et al. (2021) found that pediatricians are increasingly reporting that parents’ moral and religious concerns are a barrier to HPV vaccine uptake [27]; such perceptions may cause apprehension about discussing sexual health topics with patients and strongly recommending the HPV vaccine.

Despite these implications, some study limitations exist. First, while single-item indices of religious attendance and religious salience are commonly used to assess the relationship between religion and the HPV vaccine decision, measures that capture religious affiliation or that assess religious beliefs/teachings that influence the vaccine decision may be better suited for such inquiry. Studies show parents from conservative religious traditions may be less likely to vaccinate youth against HPV [13, 28]. Second, the survey question assessing parents’ vaccine intention did not include a ‘Maybe/Unsure’ response option, which may have forced parents who were undecided about whether they vaccinate their child at some point in the future to select an answer choice that does not truly reflect their vaccine intention. To this end, study results should be interpreted with caution; additional studies with more robust measures are needed to confirm study findings. Third, convenience sampling was used to recruit caregivers interested in parent-adolescent sexual health communication into the study. Therefore, findings that religiosity does not influence parents’ vaccine decision may be a consequence of selection bias (i.e., parents participating in the study may have been more inclined to vaccinate, or to be open to vaccinating, than those in the general population).

Despite these limitations, this study provides important contributions to the literature. As previously noted, we analyzed HPV vaccine outcomes for Black and Hispanic parents using separate models; we recognize this as a strength of the study, given the unique barriers to HPV vaccine uptake in these groups. To our knowledge, this is the first study to examine the impact of religiosity on HPV vaccine decision-making for Hispanic parents, in particular, and one of the few that examines this relationship quantitatively for urban, racial/ethnic minority parents. We believe understanding this relationship is critical, given that Black and Hispanic populations are the most religious racial/ethnic groups in the U.S. as well as the groups most impacted by HPV and HPV-associated cancers, and this study represents a first step towards that end.

In conclusion, our study suggests that religiosity is not associated with HPV vaccine decision-making for urban, Black and Hispanic parents. Future studies using measures that capture the complexity of religion as a social construct are needed to confirm the findings. In addition, studies with representative sampling will enable us to make generalizations about the influence of religion on HPV vaccine decision-making for urban, racial/ethnic minority parents.

## Data Availability

The datasets used and/or analyzed during the current study are available from the corresponding author on reasonable request.
